# Endocrinopathies Associated with Immune Checkpoint Inhibitor Cancer Treatment: A Review

**DOI:** 10.3390/jcm9072033

**Published:** 2020-06-29

**Authors:** Naoko Okura, Mai Asano, Junji Uchino, Yoshie Morimoto, Masahiro Iwasaku, Yoshiko Kaneko, Tadaaki Yamada, Michiaki Fukui, Koichi Takayama

**Affiliations:** 1 Department of Pulmonary Medicine, Graduate School of Medical Science, Kyoto Prefectural University of Medicine, Kyoto 602-8566, Japan; ku-n07@koto.kpu-m.ac.jp (N.O.); yoshie-m@koto.kpu-m.ac.jp (Y.M.); miwasaku@koto.kpu-m.ac.jp (M.I.); kaneko-y@koto.kpu-m.ac.jp (Y.K.); tayamada@koto.kpu-m.ac.jp (T.Y.); takayama@koto.kpu-m.ac.jp (K.T.); 2 Department of Endocrinology and Metabolism, Graduate School of Medical Science, Kyoto Prefectural University of Medicine, Kyoto 602-8566, Japan; maias@koto.kpu-m.ac.jp (M.A.); michiaki@koto.kpu-m.ac.jp (M.F.)

**Keywords:** immune checkpoint inhibitors, immune-related adverse events, endocrine disorders, tumor-bearing patients

## Abstract

Treatment with immune checkpoint inhibitors has shown efficacy against a variety of cancer types. The effects of nivolumab and pembrolizumab on lung cancer have been reported, and further therapeutic advances are ongoing. The side effects of immune checkpoint inhibitors are very different from those of conventional cytocidal anticancer drugs and molecular targeted drugs, and they involve various organs such as the digestive and respiratory organs, thyroid and pituitary glands, and skin. The generic term for such adverse events is immune-related adverse events (irAEs). They are relatively infrequent, and, if mild, treatment with immune checkpoint inhibitors can be continued with careful control. However, early detection and appropriate treatment are critical, as moderate-to-severe irAEs are associated with markedly reduced organ function and quality of life, with fatal consequences in some cases. Of these, endocrinopathies caused by immune checkpoint inhibitors are sometimes difficult to distinguish from nonspecific symptoms in patients with advanced cancer and may have serious outcomes when the diagnosis is delayed. Therefore, it is necessary to anticipate and appropriately address the onset of endocrinopathies during treatment with immune checkpoint inhibitors. Here, we present a review of endocrine disorders caused by immune checkpoint inhibitor treatment.

## 1. Introduction

In 2011, the Food and Drug Administration (FDA) approved the immune checkpoint inhibitor (ICI) ipilimumab, an anti-cytotoxic T-lymphocyte antigen-4 (anti-CTLA-4) antibody, for the treatment of malignant melanoma. Since then, anti-programmed cell death 1 (anti-PD-1) antibodies, such as nivolumab and pembrolizumab, and anti-programmed death ligand 1 (PD-L1) antibodies, such as atezolizumab and durvalumab, have been developed and approved for lung cancer treatment [1]. The effectiveness of cancer immunotherapy using these drugs can also be observed in cases receiving combined treatment with cytotoxic anticancer drugs and radiotherapy, and further expansion of the indications is expected in future.

CTLA-4 is a protein expressed on the surfaces of activated T-cells, and it inhibits T-cell activation after binding to antigen-presenting cells [2,3]. Anti-CTLA-4 antibodies bind to CTLA-4 and block the inhibitory receptors of activated T-cells, thus exerting antitumor effects. They also bind to the surface CTLA-4 in regulatory T-cells (Tregs), thus reducing the immunosuppressive function of Tregs and enhancing tumor immune responses [4]. Furthermore, they are thought to reduce Tregs in tumor tissue via antibody-dependent cellular cytotoxicity (ADCC) and induce tumor cell death [5,6,7].

PD-1 is expressed on the surfaces of activated T-cells, and its binding with one of its ligands, PD-L1, inhibits T-cell activation [8]. Anti-PD-1 and anti-PD-L1 antibodies block the inhibitory system’s signal to T-cells by obstructing such bindings, thus exerting antitumor effects. Therefore, while ICIs exhibit antitumor effects via a novel mechanism, immune adjustments do not work correctly in all cases, and side effects resembling autoimmune and inflammatory diseases have been reported. These types of adverse events, termed immune-related adverse events (irAEs), are characteristic side effects of ICI treatment, being distinct from the side effects of conventional anticancer drug treatment [9]. IrAEs involve almost the entire body, including the skin, gastrointestinal tract, and liver. In particular, endocrinopathy is a relatively frequent irAE [10,11].

Hypopituitarism, adrenocortical dysfunction, thyroid dysfunction, and type 1 diabetes mellitus are common endocrine disorders caused by ICI treatment [12]. Moreover, a small association between hypoparathyroidism and ICI treatment has been reported [13,14,15,16]. Pituitary dysfunction is frequent in patients receiving anti-CTLA-4 antibodies, while thyroid dysfunction is prevalent in patients receiving anti-PD-1/anti-PD-L1 antibodies [9,12]. Adrenal insufficiency is infrequent in patients treated with either drug [9,12]. Many symptoms of adrenal insufficiency, such as anorexia and malaise, are nonspecific and often observed in tumor-bearing patients. However, adrenal insufficiency can progress to fatal disease states such as adrenal crisis, and early detection and treatment according to the cause are necessary. Finally, although the frequency of type I diabetes mellitus associated with ICI treatment is small, clinicians should be aware that some cases can develop fulminant disease and should take the necessary actions in the early stages. It is important to be aware that symptoms such as fatigue caused by endocrinopathy may be misidentified as caused by the underlying cancer, and that endocrinopathy may occur with this drug when using immune checkpoint inhibitors. Table 1 shows the major ICIs indications and irAEs.

Here we present a review of endocrine disorders caused by ICI treatment.

## 2. Hypopituitarism

Hypopituitarism as an irAE is more common in patients receiving anti-CTLA-4 antibodies than in those receiving anti-PD-1/anti-PD-L1 antibodies, with the reported incidences being approximately 10% and ≤1% [17,18,19,20,21,22]. In addition, it has been reported that the incidence of hypophituitarism is higher with the concomitant use of anti-CTLA-4, and anti-PD-1/PD-L1 antibodies are more common than the use of a single agent [12]. Hypopituitarism caused by ICI treatment is classified into hypophysitis and isolated adrenocorticotropic (ACTH) deficiency. Pituitary gland enlargement is seen in hypophysitis, which causes hyposecretion of several anterior pituitary hormones, including thyroid-stimulating hormone (TSH), gonadotropins, and ACTH. On the other hand, the pituitary gland does not enlarge in ACTH deficiency, wherein the secretory capacity of only ACTH is reduced. There are very few reports of posterior pituitary dysfunction [17,23]. Although both patterns of dysfunction can occur in patients receiving anti-CTLA-4 antibodies, the use of anti-PD-1/anti-PD-L1 antibodies has been associated with ACTH deficiency in most cases [17,20,24]. Hypopituitarism often develops 4–10 weeks after treatment initiation due to anti-CTLA-4 antibodies [12]. An association between the incidence and the dose has also been noted, with one report showing a two-fold higher incidence in patients receiving high-dose ipilimumab (10 mg/kg) than in those receiving low-dose ipilimumab (3 mg/kg) [19]. Moreover, the higher dose (10 mg/kg) resulted in more adverse events than did the lower dose (3 mg/kg). However, significantly longer survival associated with the higher dose has been documented in some reports, and an association between irAE development and treatment efficacy has been pointed out [18,24]. Hypopituitarism also occurs within months to 1 year after treatment initiation due to anti-PD-1/anti-PD-L1 antibodies, and it may even develop after discontinuation of the drug [25,26,27]. It should be noted that ACTH hyposecretion always develops in all cases of hypopituitarism due to ICI treatment. The symptoms of ICI-induced hypopituitarism include anorexia and malaise due to secondary adrenal insufficiency, weight loss, gastrointestinal symptoms (nausea, vomiting, and diarrhea), hypotension, and hypoglycemia. In addition, headache, visual field impairment, and visual impairment occur in cases of hypophysitis with high-grade enlargement of the pituitary gland. In blood examination, abnormal findings such as hyponatremia and eosinophilia are recognized.

If hypopituitarism is suspected, it is necessary to measure the hormones secreted by the anterior pituitary gland and target organs. With regard to hypophysitis, diffuse enlargement and swelling of the pituitary gland and pituitary stalk with enhancement on contrast-enhanced magnetic resonance imaging are observed in more than half of the cases [17]. Subsequently, the enlarged pituitary gland gradually shrinks in the acute phase, and pituitary function is partially or completely lost [28,29]. During long-term observations, (median follow-up, 33 months) in one study, many of the thyroid and gonadal dysfunctions were found to be reversible, whereas ACTH hyposecretion was irreversible in most cases [29]. The pathogenesis of hypopituitarism due to ICI treatment remains unclear. In autopsied cases of pituitary dysfunction caused by tremelimumab, anti-CTLA-4 antibody, necrotic changes, and lymphocytic infiltrates with fibrosis were observed in the anterior pituitary gland. In addition, complement component 4 fragment (C4d) deposition associated with complement activation was observed; this suggested the involvement of both type IV and type II allergic reactions [17].

## 3. Adrenal Insufficiency

Adrenal insufficiency caused by ICI treatment includes primary and secondary adrenal insufficiency caused by hypopituitarism. Most cases are considered to have secondary adrenal insufficiency, and primary adrenal insufficiency is thought to be less frequent, with a reported incidence of 1.4% (95% confidence interval (CI): 0.9–2.2) for ipilimumab, 2.0% (95% CI: 0.9–4.3) for nivolumab, and 5.2% (95% CI: 2.9–9.2) to 7.6% (95% CI: 1.2–36.8) for nivolumab or pembrolizumab combined with ipilimumab [12]. The time of onset is estimated as one to several months after the start of treatment [22,30]. The symptoms of adrenal insufficiency are nonspecific and include fatigue, anorexia, abdominal pain, nausea, weight loss, hypotension, and hypoglycemia. The appearance of hyponatremia, eosinophilia, and neutropenia suggests the development of adrenal insufficiency. A low morning serum cortisol level despite an elevated plasma ACTH level suggests primary adrenal insufficiency, whereas a low plasma ACTH level suggests secondary adrenal insufficiency. Serum cortisol levels of ≥18 μg/dL are considered to indicate the absence of adrenal dysfunction, while adrenal dysfunction is represented by serum cortisol levels of <4 μg/dL. When the serum cortisol level is ≥4 μg/dL and <18 μg/dL, a rapid ACTH tolerance test or an insulin-hypoglycemia test can confirm the diagnosis [31]. Bilateral adrenal enlargement on abdominal computed tomography (CT) and fluorodeoxyglucose (FDG) uptake in the bilateral adrenal glands on positron emission tomography (PET) have been reported; however, similar findings may be observed in cases of adrenal metastasis, which warrant careful judgment [32]. With regard to the pathogenesis of primary adrenal insufficiency caused by ICI treatment, adrenal autoantibodies have been detected in one case of pembrolizumab-induced adrenal insufficiency, although several points remain to be clarified [31].

## 4. Thyroid Dysfunction

Among endocrinopathies occurring as irAEs, thyroid dysfunction is the most frequent. Although thyroid dysfunction is mainly caused by thyrotoxicosis, while hypothyroidism is caused by destructive thyroiditis, the occurrence of Basedow’s disease after the administration of anti CTLA-4 antibodies has also been reported [33,34]. The reported incidence of thyroid dysfunction with the use of anti-PD-1 antibodies is 5–10%. The incidence of thyroid dysfunction is higher with the use of anti-PD-L1 antibodies (0–5%) and anti-CTLA-4 antibodies (0–5%) [19,20,21]. In a previous systematic review and meta-analysis, increased use of combination treatment was observed, with hypothyroidism occurring in 16.4% cases treated with nivolumab combined with ipilimumab [12]. Moreover, the incidence of hypothyroidism was significantly higher with ICI treatment than with chemotherapy and placebo treatment [12]. In other studies, thyroid dysfunction was more frequent in cases treated with anti-thyroglobulin (Tg) antibody (anti-TgAb) and anti-thyroid peroxidase antibody (anti-TPOAb) than in cases treated without these antibodies before nivolumab treatment initiation; this finding may be beneficial for predicting the onset of thyroid dysfunction [35,36]. Destructive thyroiditis occurs within a few weeks after ICI treatment initiation in many cases, and it may present with thyrotoxicosis [37,38,39]. Subsequently, patients may exhibit a transition to hypothyroidism within 3–6 weeks [37,38,39,40]. In a previous study, 12 of 99 patients who received pembrolizumab developed thyrotoxicosis, and transition to hypothyroidism occurred in nine of the 12 patients [39]. Another study found thyrotoxicosis in six of 10 patients who developed indolent thyroiditis after pembrolizumab treatment initiation; all six patients exhibited a transition to hypothyroidism after four weeks [38].

Symptoms of thyrotoxicosis include palpitations, sweating, fever, diarrhea, tremors, weight loss, and general fatigue. Neck pain is generally not observed. Blood tests show a decreased serum TSH level, elevated serum free T3 (FT3) and free T4 (FT4) levels, and negativity for thyroid receptor antibody (TRAb). Quite often, anti-TgAb and anti-TPOAb are positive [30,36]. In addition, there are many cases in which the increased serum Tg level is recognized by destruction of the thyroid gland, and it is said that the Tg level normalizes upon transitioning to hypothyroidism [40]. Thyroid echography frequently shows decreased blood flow and an internal heterogeneous low signal intensity. FDG-PET also shows increased uptake, while thyroid scintigraphy shows decreased iodine uptake [38].

Hypothyroidism may develop after thyrotoxicosis or simultaneously with the onset of thyroiditis. If the latter occurs, positivity for anti-TgAb and anti-TPOAb is seen in several cases [37]. Major symptoms include general fatigue, loss of appetite, constipation, bradycardia, and weight gain. Blood tests show elevated serum TSH and decreased serum FT4 and FT3 levels. In mild cases, a slightly high TSH level may result in a state of occult hypothyroidism. Thyroid echography may show decreased blood flow, parenchymal hypointensity, and atrophy. Hypothyroidism secondary to hypopituitarism must be ruled out in patients showing hypothyroidism. Hypopituitarism can be suspected when serum FT4 levels are low and TSH levels are low to normal. Differentiation should be cautious because low serum FT3 levels also occur in the end stages of malignancy and in low T3 syndrome complicating severe infections. In low T3 syndrome, the serum FT3 level is low and the serum FT4 level is normal or slightly decreased, while the serum TSH level is normal.

The mechanism by which ICIs cause thyroid dysfunction has not been clarified, but it has been suggested that the expression of PD-L1 and programmed death ligand 2 in the thyroid tissue plays a role [41].

## 5. Type 1 Diabetes Mellitus

Type 1 diabetes induced by ICI treatment results from the destruction of β-cells by ICIs and is reportedly more frequent with the use of anti-PD1/anti-PD-L1 antibodies. However, the reported incidences are 2.0% and 0.4% with nivolumab and pembrolizumab; thus, it seems to be a less common irAE [12]. The incidence of type I diabetes after ipilimumab treatment is even lower [20,42]. However, fulminant type I diabetes mellitus can worsen rapidly and prove fatal, and it is necessary to consider the possibility of this complication when administering ICIs. The time from anti-PD-1 therapy initiation to the onset of type 1 diabetes has been reported to be 13–504 days [43]. Symptoms include dry mouth, polydipsia, and polyuria due to hyperglycemia in mild-to-moderate cases. Severe disease is associated with ketosis, ketoacidosis, general fatigue, and disturbed consciousness, with further progression resulting in coma.

Fulminant type I diabetes mellitus exhibits a hyperacute onset over several days, and endogenous insulin is depleted at the time of diagnosis [44]. On the other hand, type I diabetes mellitus develops relatively slowly over the course of several weeks.

Because β-cell dysfunction is generally irreversible, it is important to make a diagnosis and initiate treatment before the development of ketoacidosis. Diabetes mellitus should be suspected when symptoms of hyperglycemia appear and fasting blood sugar and random blood sugar levels exceed 126 and 200 mg/mL, respectively. In such cases, definite diagnosis and diagnosis of the disease type should be performed.

Blood glucose levels may be elevated to approximately 200–300 mg/dL, although elevation to approximately 1000 mg/dL is also possible. The HbA1c level is also elevated, notwithstanding it is lower relative to the blood glucose level. The C peptide level gradually decreases in serum and urine, and anti-glutamic acid decarboxylase antibodies are generally absent.

## 6. Treatment

### 6.1. Hypopituitarism

Table 2 presents management strategies for hypopituitarism according to the Common Terminology Criteria for Adverse Events (CTCAE) grade. Treatment generally involves hormone replacement therapy. ACTH deficiency is treated with hydrocortisone (10 to 20 mg/day). High doses of glucocorticoids have been reported to improve pituitary enlargement [24,45]. On the other hand, it has been reported that high doses of glucocorticoids do not contribute to restoration of the secretory capacity of ACTH and are associated with relatively high mortality [24,45]. High-dose glucocorticoids are recommended only if the condition is associated with headache and pituitary enlargement with visual field damage. When both TSH and ACTH secretion disorders are present, hydrocortisone replacement therapy must be preceded by hormone replacement therapy. The use of ICIs in patients with treatment-induced hypopituitarism should be discontinued until treatment stabilizes their general condition.

### 6.2. Adrenal Insufficiency

Table 3 presents management strategies for adrenal insufficiency according to the CTCAE grade. The condition should be managed according to its severity. Hydrocortisone (10–20 mg/day) replacement therapy should be initiated for patients with only laboratory abnormalities or mild symptoms that permit activities of daily living [46]. In case of adrenal crisis, systemic management and early administration of hydrocortisone are necessary. In all cases, consultation with an endocrinologist is recommended for medical care. If primary adrenal insufficiency due to ICI treatment occurs, the drugs should be discontinued and administered after stabilization of the patient’s general condition by treatment.

### 6.3. Thyroid Dysfunction

Table 4 and Table 5 show the management strategies for hyperthyroidism and hypothyroidism, respectively, according to the CTCAE grade. For thyrotoxicosis caused by destructive thyroiditis, antithyroid drugs are not necessary because the duration of symptoms is usually short. When symptoms such as tremors and motivation are recognized, symptomatic treatment with a β-blocker is required. Antithyroid drugs are reserved for patients with Basedow’s disease.

In case of hypothyroidism, if the TSH level is <10 mIU/L and no symptoms are observed, ICI administration is continued and serum TSH, FT4, and FT3 levels are monitored. If the TSH level is ≥10 mIU/L and moderate symptoms are present, thyroid hormone replacement therapy is planned [37]. In case of concomitant adrenal insufficiency, careful monitoring is required, and thyroid hormone replacement should be preceded by the administration of hydrocortisone if the adrenal insufficiency worsens.

ICI treatment can be resumed when treatment with or without thyroid hormone replacement therapy results in amelioration of symptoms.

### 6.4. Type 1 Diabetes Mellitus

Insulin therapy is the mainstay of treatment for type 1 diabetes mellitus due to ICI treatment, and immediate treatment must be initiated. If ketosis or ketoacidosis is present, immediate-acting insulin should be continuously administered, along with intravenous saline infusion and electrolyte management. After the ketosis or ketoacidosis has improved, the patient can be switched to insulin therapy. Once insulin treatment reduces the blood glucose levels, ICI treatment can be resumed.

## 7. Adrenal Insufficiency in Tumor-Bearing Patients

As noted above, primary/secondary adrenal insufficiency is a less common but potentially fatal irAE, and it often includes adrenal crisis, in patients receiving ICIs. On the other hand, symptoms are often nonspecific, such as anorexia and malaise, which are also common symptoms in cancer patients. Adrenal insufficiency is also a common condition in cancer patients, and efforts must be made to detect it at an early stage.

Causes of adrenal insufficiency other than ICI treatment in cancer patients include steroid withdrawal syndrome, adrenal metastases from primary disease, and autoimmune adrenalitis.

Long-term corticosteroid treatment may be used for various purposes in cancer patients, including palliation of symptoms such as fatigue, resolution of cerebral edema, and treatment of drug-induced or radioactive organ damage. In addition, they are often administered during anticancer drug therapy. Long-term corticosteroid use causes hypothalamic–pituitary–adrenal suppression and adrenal atrophy. Steroid withdrawal syndrome may occur when steroids are suddenly reduced or discontinued, and many patients present with clinical features of acute adrenal insufficiency. It is necessary to pay attention to sudden dose reduction and discontinuation in patients who have been receiving long-term steroid treatment, and steroid withdrawal syndrome should be suspected when symptoms indicating adrenal insufficiency are observed. In case of steroid withdrawal syndrome, the symptoms rapidly disappear when the steroid dose is increased in most cases.

Moreover, when physical stresses such as diarrhea, trauma, and dehydration occur in patients receiving long-term corticosteroid treatment, relative steroid deficiency may develop and result in adrenal insufficiency symptoms. The causative disease should be treated, and the dose of the steroid drug should be increased. Failure to take appropriate measures may result in adrenal crisis and potentially fatal conditions. On the other hand, in cancer patients, metastatic adrenal tumors may cause adrenal insufficiency. A previous study involving autopsy of malignant tumors found adrenal metastasis in approximately 3% cases [47]. Further, chronic primary adrenocortical insufficiency due to metastatic adrenal tumors is rare and has been reported to occur in approximately 1% patients [47]. Even in cases of metastatic adrenal tumors, cortisol secretion is preserved until approximately 90% of the bilateral adrenal glands are destroyed, and the typical symptoms may not appear in many cases, which complicates diagnosis [48].

## 8. Adrenal Crisis

Adrenal crisis can occur when infection and injury are complicated by adrenocortical insufficiency, and it progresses to a fatal disease state via absolute and relative steroid deficiency. Primary/secondary adrenal insufficiency due to ICI treatment may also lead to adrenal crisis, and early diagnosis and appropriate measures should be implemented at onset. The initial symptoms of adrenal crisis, like those of adrenal insufficiency, are nonspecific and include general malaise, anesthesia, loss of appetite, weight loss, nausea, abdominal pain, and fever. However, after >12 h, consciousness disturbance and hypotension can occur.

Blood tests often show hyponatremia, hyperkalemia, hypoglycemia, dehydration, and eosinophilia. When adrenal crisis is suspected on the basis of the medical history and test results, immediate measures should be taken while excluding other conditions such as sepsis. Initial treatment includes infusion of a large volume of saline, glucose solution, and hydrocortisone. Measurements of blood cortisol and ACTH are useful for diagnosis.

## 9. Conclusions

**Figure 1:**
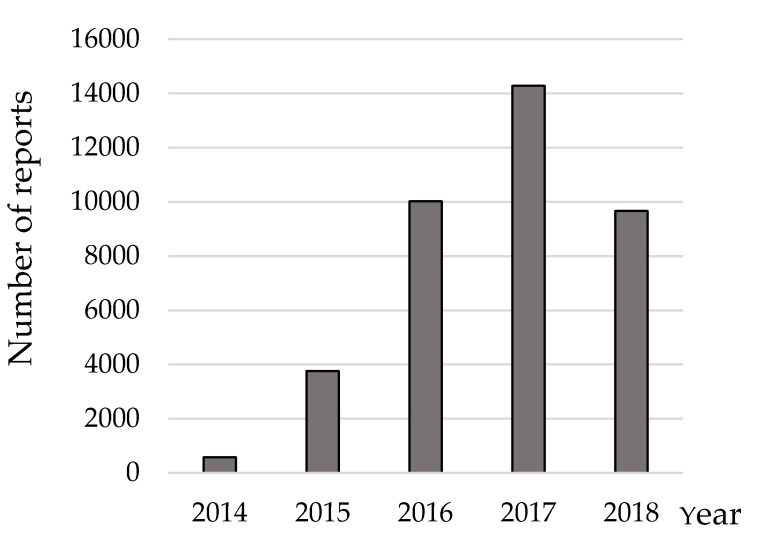
Adopted from Reference [49]. Food and Drug Administration (FDA)-reported numbers of immune-related Adverse Events (irAEs) with anti programmed cell death 1 (PD-1)/ programmed death ligand 1 (PD-L1) antibody monotherapy versus anti PD-1/PD-L1 antibody plus anti cytotoxic T-lymphocyte antigen-4 (CTLA-4) antibody combination treatment. (Number of reports up to June in 2018).

In summary, endocrine dysfunction is a frequent irAE associated with ICI treatment. Anti-CTLA-4 antibodies often cause hypopituitarism, while anti-PD-1/anti-PD-L1 antibodies cause thyroid dysfunction. Primary adrenal insufficiency and type I diabetes mellitus are less frequent with all ICIs. Hypopituitarism may also cause secondary adrenal insufficiency via ACTH hyposecretion. Symptoms of adrenal insufficiency are nonspecific and common also in cancer patients; therefore, diagnosis may be difficult. Moreover, symptoms of adrenal insufficiency in cancer patients often have a background other than irAE caused by ICI in tumor bearing patients. While adrenal insufficiency leads to adrenal crisis in severe cases, type 1 diabetes mellitus may progress to fulminant disease; thus, both conditions should be detected and treated at the early stages. As the indications of ICIs expand, the number of irAEs episodes also tends to increase as shown in Figure 1. In the future, early detection and proper management of endocrine dysfunction should be considered important for the treatment using ICI as mentioned above.

## Figures and Tables

**Table 1:** Indication and major endocrinopathies of immune checkpoint inhibitors (ICIs).

Target	Drug	Indication	Major Endocrinopathies
Anti CTLA-4 antibody	Ipilimumab	Malignant melanoma	Hypopituitarism
		Renal cell cancer	
Anti-PD-1 antibody	Nivolumab	Malignant melanoma	Hypothyroidism Hyperthyroidism
		Non-small cell lung cancer	
		Renal cell cancer	
		Hodgkin lymphoma	
		Head and neck cancer	
		Gastric cancer	
		Malignant mesothelioma	
		Colorectal cancer with high-frequency microsatellite instability (MSI-High)	
		Esophageal cancer	
	Pembrolizumab	Non-small cell lung cancer	Hypothyroidism Hyperthyroidism
		Hodgkin lymphoma	
		Urothelial cancer	
		Solid cancers with high-frequency microsatellite instability (MSI-High)	
		Renal cell cancer	
		Head and neck cancer	
Anti PD-L1 antibody	Atezolizumab	Non-small cell lung cancer	Hypothyroidism
		Small cell lung cancer	
		Breast cancer	
	Durvalumab	Non-small cell lung cancer	Hypothyroidism
			Hyperthyroidism
	Avelumab	Merkel cell carcinoma renal cell cancer	Hypothyroidism

PD-1, programmed cell death 1; PD-L1, programmed death ligand 1; CTLA-4, cytotoxic T-lymphocyte antigen-4.

**Table 2:** Management of hypopituitarism induced by immune checkpoint inhibitors.

CTCAE Grade	Management	Treatment of Adverse Event
Grade 1	Hormone supplementation as needed	Consider consultation with an endocrinologist If adrenal insufficiency is suspected, start hydrocortisone 10–20 mg BID Start testosterone or estrogen replacement therapy if needed
Grade 2	Stop ICI treatment until symptoms stabilize by hormone supplementation After amelioration of symptoms, resume administration of ICI	Consult an endocrinologist Consider pituitary imaging Perform hormone replacement therapy as performed for Grade 1 events Perform frequent thyroid function and other hormonal tests until baseline levels are achieved
Grade 3	Same as above	Consult an endocrinologist Consider pituitary imaging Perform a pituitary function test on hospitalization If adrenal insufficiency is present, start hydrocortisone 15–30 mg BID Perform hormone replacement therapy as performed for Grade 1 events Perform frequent thyroid function and other hormonal tests until baseline levels are achieved
Grade 4	Stop ICI treatment Resume administration after recovery from crisis and stabilization of symptoms	Perform full-body management during hospitalization Consult an endocrinologist Immediately start administration of hydrocortisone 100–200 mg BID Physiological saline infusion under cardiac function monitoring Consider pituitary imaging Perform frequent thyroid function and other hormonal tests until baseline levels are achieved

CTCAE, Common Terminology Criteria for Adverse Events; ICI, immune checkpoint inhibitor; BID, bis in die

**Table 3:** Management of adrenal insufficiency induced by immune checkpoint inhibitors.

CTCAE Grade	Management	Treatment of Adverse Event
Grade 1	Hormone supplementation as needed After amelioration of symptoms, resume administration of ICI	Consult an endocrinologist If adrenal insufficiency is suspected, start hydrocortisone 10–20 mg BID
Grade 2	Stop ICI treatment until symptoms stabilize by hormone supplementation After amelioration of symptoms, resume administration of ICI	Consult an endocrinologist Perform hormone replacement therapy as performed for Grade 1 events Perform frequent hormonal tests until baseline levels are achieved
Grade 3	Same as above	Consult an endocrinologist Perform an adrenal function test on hospitalization If adrenal insufficiency is present, start hydrocortisone 15–30 mg BID
Grade 4	Stop ICI treatment Resume administration after recovery from crisis and stabilization of symptoms	Perform full-body management during hospitalization Consult an endocrinologist Immediately start administration of hydrocortisone 100–200 mg BID Physiological saline infusion under cardiac function monitoring Perform an adrenal function test after the general condition has stabilized

CTCAE, Common Terminology Criteria for Adverse Events; ICI, immune checkpoint inhibitor.

**Table 4:** Management of hyperthyroidism induced by immune checkpoint inhibitors.

CTCAE Grade	Management	Treatment of Adverse Event
Grade 1	Continue ICI treatment	Continue to monitor TSH and FT4 levels until hyperthyroidism disappears
Grade 2	Stop ICI treatment until the symptoms ameliorate or test values become normal After amelioration of symptoms, resume administration of ICI	Consult an endocrinologist Perform a thyroid function test every 2–3 weeks If the thyroid poisoning does not resolve after 6–8 weeks, Graves’ disease is differentiated
Grade 3 or 4	Same as above	Consult an endocrinologist Start administration of β-blocker Conduct clinical tests every 1–3 weeks In case of thyroid crisis, treat the patient in the intensive care unit

CTCAE, Common Terminology Criteria for Adverse Events; ICI, immune checkpoint inhibitor; TSH, thyroid-stimulating hormone; FT4, free T4.

**Table 5:** Management of hypothyroidism induced by immune checkpoint inhibitors.

CTCAE Grade	Management	Treatment of Adverse Event
Grade 1	Continue ICI treatment	Continue to monitor TSH, FT3, and FT4 levels every 2–3 weeks
Grade 2	Stop ICI treatment until the symptoms ameliorate or test values become normal After amelioration of symptoms, resume administration of ICI	Consult an endocrinologist Start thyroid hormone replacement therapy if symptoms are present or the TSH level is high If thyroid function is stable, perform a thyroid function test every 6 weeks
Grade 3 or 4	Same as above	Consult an endocrinologist Start administration of β-blocker In case of myxedema coma, treat the patient in the intensive care unit Following stabilization of symptoms, treat as per the protocol for Grade 2 events

CTCAE, Common Terminology Criteria for Adverse Events; ICI, immune checkpoint inhibitor; TSH, thyroid-stimulating hormone; FT4, free T4; FT3, free T3.
